# The type I interferon signature in leukocyte subsets from peripheral blood of patients with early arthritis: a major contribution by granulocytes

**DOI:** 10.1186/s13075-016-1065-3

**Published:** 2016-07-13

**Authors:** Tamarah D. de Jong, Joyce Lübbers, Samina Turk, Saskia Vosslamber, Elise Mantel, Hetty J. Bontkes, Conny J. van der Laken, Johannes W. Bijlsma, Dirkjan van Schaardenburg, Cornelis L. Verweij

**Affiliations:** Department of Pathology, VU University Medical Center, Amsterdam, The Netherlands; Amsterdam Rheumatology and Immunology Center, VU University Medical Center, Amsterdam, The Netherlands; Amsterdam Rheumatology and Immunology Center, VU University Medical Center, Reade, Amsterdam, The Netherlands; Present address: Department of Oral Cell Biology, Academisch Centrum Tandheelkunde Amsterdam, Amsterdam, The Netherlands; Amsterdam Rheumatology and Immunology Center, VU University Medical Center, Academic Medical Center, Amsterdam, The Netherlands

**Keywords:** Type I interferon, Rheumatoid arthritis, Granulocytes

## Abstract

**Background:**

The type I interferon (IFN) signature in rheumatoid arthritis (RA) has shown clinical relevance in relation to disease onset and therapeutic response. Identification of the cell type(s) contributing to this IFN signature could provide insight into the signature’s functional consequences. The aim of this study was to investigate the contribution of peripheral leukocyte subsets to the IFN signature in early arthritis.

**Methods:**

Blood was collected from 26 patients with early arthritis and lysed directly or separated into peripheral blood mononuclear cells (PBMCs) and polymorphonuclear granulocytes (PMNs). PBMCs were sorted into CD4^+^ T cells, CD8^+^ T cells, CD19^+^ B cells, and CD14^+^ monocytes by flow cytometry. Messenger RNA expression of three interferon response genes (IRGs *RSAD2*, *IFI44L*, and *MX1*) and type I interferon receptors (*IFNAR1* and *IFNAR2*) was determined in whole blood and blood cell subsets by quantitative polymerase chain reaction. IRG expression was averaged to calculate an IFN score for each sample.

**Results:**

Patients were designated “IFN^high^” (*n* = 8) or “IFN^low^” (*n* = 18) on the basis of an IFN score cutoff in whole peripheral blood from healthy control subjects. The difference in IFN score between IFN^high^ and IFN^low^ patients was remarkably large for the PMN fraction (mean 25-fold) compared with the other subsets (mean 6- to 9-fold), indicating that PMNs are the main inducers of IRGs. Moreover, the relative contribution of the PMN fraction to the whole-blood IFN score was threefold higher than expected from its abundance in blood (*p* = 0.008), whereas it was three- to sixfold lower for the other subsets (*p* ≤ 0.063), implying that the PMNs are most sensitive to IFN signaling. Concordantly, *IFNAR1* and *IFNAR2* were upregulated compared with healthy controls selectively in patient PMNs (*p* ≤ 0.0077) but not in PBMCs.

**Conclusions:**

PMNs are the main contributors to the whole-blood type I IFN signature in patients with early arthritis, which seems due to increased sensitivity of these cells to type I IFN signaling. Considering the well-established role of neutrophils in the pathology of arthritis, this suggests a role of type I IFN activity in the disease as well.

**Electronic supplementary material:**

The online version of this article (doi:10.1186/s13075-016-1065-3) contains supplementary material, which is available to authorized users.

## Background

Rheumatoid arthritis (RA) manifests as a heterogeneous disease with a clinical spectrum ranging from mild to severe disease. This heterogeneity most likely has its origin in the multifactorial nature of the disease, whereby specific combinations of genetic risk factors, together with an appropriate environmental trigger, influence not only susceptibility but also the severity, pathogenesis, and therapeutic outcome.

Heterogeneity of RA is partly reflected at the level of gene expression. Genome-wide gene expression analysis revealed evidence for molecular differences between patients with RA, in particular in the type I interferon (IFN) response gene program [1]. Some of the patients with RA display a so-called IFN signature, which is characterized by relatively high expression of type I IFN response genes (IRGs). Induction of these IRGs is triggered via activation of the type I interferon α/β receptors *IFNAR1* and *IFNAR2*, which dimerize and subsequently activate the Janus kinase-signal transducer and activator of transcription (JAK-STAT) signaling pathway, more specifically JAK1, TYK2, STAT1, and STAT2, eventually resulting in recruitment of IRF9 and formation of the ISGF3 transcription factor complex [2]. Although the presence of the IFN signature in RA is not found to be associated with disease parameters such as disease activity or presence of rheumatoid factor and/or anticitrullinated protein antibodies (ACPA) [3], several studies have demonstrated that the IFN signature in RA does have potential clinical relevance.

The presence of the IFN signature was shown to be a risk factor for arthritis development in preclinical disease [4, 5]. In later phases of the disease, the presence of an IFN signature was found to be associated with clinical response to different treatment regimens, such as rituximab [6–8] and tocilizumab [9]. Furthermore, type I IRG expression appears to be differentially regulated between responders and nonresponders during treatment with rituximab and anti-tumor necrosis factor (anti-TNF) therapy [10–12].

Peripheral blood is an easily accessible source for biomarker identification, and the studies mentioned above demonstrate that the peripheral blood of patients with RA reflects pathogenic processes related to the disease. However, the peripheral blood consists of several cell types, and consequently the transcriptomic profile is an accumulation of all gene expression programs that are induced in these cell types. Identification of the cell type(s) contributing to the IFN signature could provide insight into the signature’s functional consequences and potentially into personalized treatment strategies.

The aim of the present study was to investigate the contribution of the major leukocyte subsets to the IFN signature in whole blood from patients with early arthritis. Using this patient group allowed us to study the IFN signature without interference of treatment with immune-modulatory drugs that are known to affect type I IFN signaling, such as glucocorticoids or hydroxychloroquine [13–15].

## Methods

### Patient recruitment and blood collection

Patients (*n* = 26) were consecutively recruited from the early arthritis cohort within the Amsterdam Rheumatology and Immunology Center, Reade, Amsterdam, The Netherlands. Inclusion criteria were presence of at least one arthritic joint, disease duration <6 months, and no previous use of disease-modifying antirheumatic drugs or biologics. The majority of patients (81 %) fulfilled the 2010 American College of Rheumatology criteria for the classification of RA [16]. The remaining five patients were diagnosed with seronegative RA (*n* = 4) and monoarthritis (*n* = 1), according to the rheumatologist’s assessment. Healthy control subjects (*n* = 25) were recruited at the VU University Medical Center, Amsterdam. From each donor, approximately 20 ml of blood was collected by venipuncture into heparin tubes and a PAXgene tube (PreAnalytiX, Hombrechtikon, Switzerland). The PAXgene tube was stored at −20 °C until further processing. The heparinized blood was processed on the same day it was drawn. This study was approved by the medical ethics committee of VU University Medical Center and Reade, Amsterdam, The Netherlands, and informed consent was obtained from all donors.

### Peripheral blood mononuclear cell isolation and polymorphonuclear granulocyte isolation

Peripheral blood mononuclear cells (PBMCs) were isolated from heparinized blood by density gradient centrifugation using Lymphoprep (Axis-Shield, Oslo, Norway), according to the manufacturer’s protocol. PBMCs were washed, and a minimum of 1 × 10^6^ PBMCs was directly lysed in 350 μl of Buffer RLT (QIAGEN Benelux BV, Venlo, The Netherlands). A minimum of 7 × 10^6^ PBMCs was resuspended in PBS containing 1 % bovine serum albumin for subsequent flow cytometric cell sorting. Polymorphonuclear leukocytes (granulocytes; PMNs) were isolated from the remaining erythrocyte/PMN pellet by lysing the erythrocytes with Buffer EL (QIAGEN Benelux BV) as described previously [17, 18], according to the manufacturer’s protocol. The remaining PMN-enriched pellet was washed with PBS and lysed in 350 μl of Buffer RLT. Buffer RLT lysates were stored at −20 °C until RNA isolation.

### Flow cytometry and cell sorting

Absolute numbers and percentages of monocytes and lymphocyte subsets were determined using flow cytometry (BD FACSCalibur; BD Biosciences, San Jose, CA, USA) with whole heparinized blood. Quantification beads (BD Trucount; BD Biosciences) in combination with fluorescein isothiocyanate (FITC)-conjugated anti-CD45, phycoerythrin (PE)-conjugated anti-CD14, peridinin chlorophyll (PerCP)-conjugated anti-CD3, and allophycocyanin (APC)-conjugated anti-CD19 were used to measure absolute numbers of lymphocytes, monocytes, T cells, and B cells, according to the manufacturer’s instructions (all from BD Biosciences). For the T-cell subsets, anti-CD45 and anti-CD3 were taken in combination with PE-conjugated anti-CD8 and APC-conjugated anti-CD4 (all from BD Biosciences).

The following antibodies were used for the cell-sorting procedure (all from BD Biosciences): Pacific Blue-conjugated or Horizon™ V450-conjugated anti-CD3, PE-conjugated anti-CD4, FITC-conjugated anti-CD8, APC-conjugated anti-CD19, and PerCP-conjugated anti-CD14. Labeled cells were analyzed and separated using FACSAria and FACSDIVA software 6.1.3 (BD Biosciences). The nozzle size was 70 μm, and the sorting speed was 3000–5000 cells/second. For sorting purposes, a gate was set around lymphocytes, and subsequent gates were set for CD3^+^CD4^+^ T helper cells, CD3^+^CD8^+^ cytotoxic T cells, and CD19^+^ B cells, based on positivity of the markers. Monocytes were gated on the basis of forward and side scatter properties as well as CD14 positivity. From each population, a minimum of 3 × 10^5^ cells was sorted and subsequently spun down at 400 × *g* for 5 minutes, lysed in 350 μl of Buffer RLT, and stored at −20 °C until RNA isolation. Sorting purity was >90 % for 95 of 104 sorted samples. Three sorted samples, two CD19-enriched fractions, and one CD14-enriched fraction were excluded due to purities <80 %.

### RNA isolation and complementary DNA synthesis

RNA was isolated from the cell lysates and PAXgene tubes using the RNeasy Micro or Mini kit (QIAGEN Benelux BV) or the PAXgene RNA isolation kit (PreAnalytiX), respectively, according to the manufacturers’ protocols. In both procedures, a DNase (QIAGEN Benelux BV) step was included to remove any genomic DNA. RNA quantity and purity were determined using a NanoDrop spectrophotometer (Nanodrop Technologies, Wilmington, DE, USA). Either 50 ng (cell fractions) or 250 ng (PAXgene whole blood) of RNA was used for complementary DNA (cDNA) synthesis, which was performed using the RevertAid H Minus cDNA Synthesis Kit (Thermo Scientific, Waltham, MA, USA), according to the manufacturer’s protocol. Two CD19-enriched samples were excluded because of low RNA yield.

### Quantitative polymerase chain reaction and calculation of the IFN score

We determined the messenger RNA (mRNA) expression of three IFN response genes (IRGs *IFI44L*, *MX1*, and *RSAD2*) that were previously described to be components of the IFN signature in RA [1, 7, 10], and thus believed to reflect the type I IFN response in peripheral blood. IRG mRNA expression was measured on cDNA by quantitative polymerase chain reaction (qPCR). qPCR was performed using SYBR Green (Applied Biosystems, Foster City, CA, USA) and the ABI Prism 7500 HT Sequence Detection System (Applied Biosystems), according to the manufacturer’s protocols. Primers were designed using Primer Express software and guidelines (Applied Biosystems), and they are listed in Additional file 1: Table S1. To calculate arbitrary values of mRNA levels and to correct for differences in primer efficiencies, a standard curve was constructed. Expression levels of target genes were calculated relative to housekeeping gene *GAPDH*. Expression levels of the IRGs were highly correlative for all studied cell fractions (*r* ≥ 0.708, *p* < 0.0001); therefore, an IFN score was calculated by averaging the expression levels of all three genes for each sample. The presence of a type I IFN signature (referred to as IFN^high^) was defined as an IFN score above mean + 2*SD in healthy control subjects. Each IRG was also analyzed individually, which yielded results comparable to those described below (data not shown).

### Statistical analysis and calculation of expected and observed contributions

All analyses were performed using the Mann-Whitney *U* test in Prism 5 software (GraphPad Software, La Jolla, CA, USA). In order to study the relative contribution of each cell type to the whole-blood IFN signature, we calculated an “expected” and “observed” IFN score contribution. The expected contribution was based only on the distribution of the cell types in the blood and assumed that each cell type would contribute equally to the whole-blood IFN signature. For example, for a whole-blood sample with an IFN score of 2.5 that contained 3.3 % monocytes, the expected contribution of the monocytes would be 2.5 × 0.033 = 0.0825. The observed contribution was the IFN score as it was measured in a sorted cell subset, corrected for the abundance of this subset in whole blood. For example, if the sample described above had an IFN score of 3.5 in the CD14-enriched fraction, the observed IFN score contribution of the monocytes would be 3.5 × 0.033 = 0.1155.

## Results

### Patients’ characteristics and selection of IFN^high^ and IFN^low^ patients

**Fig. 1:**
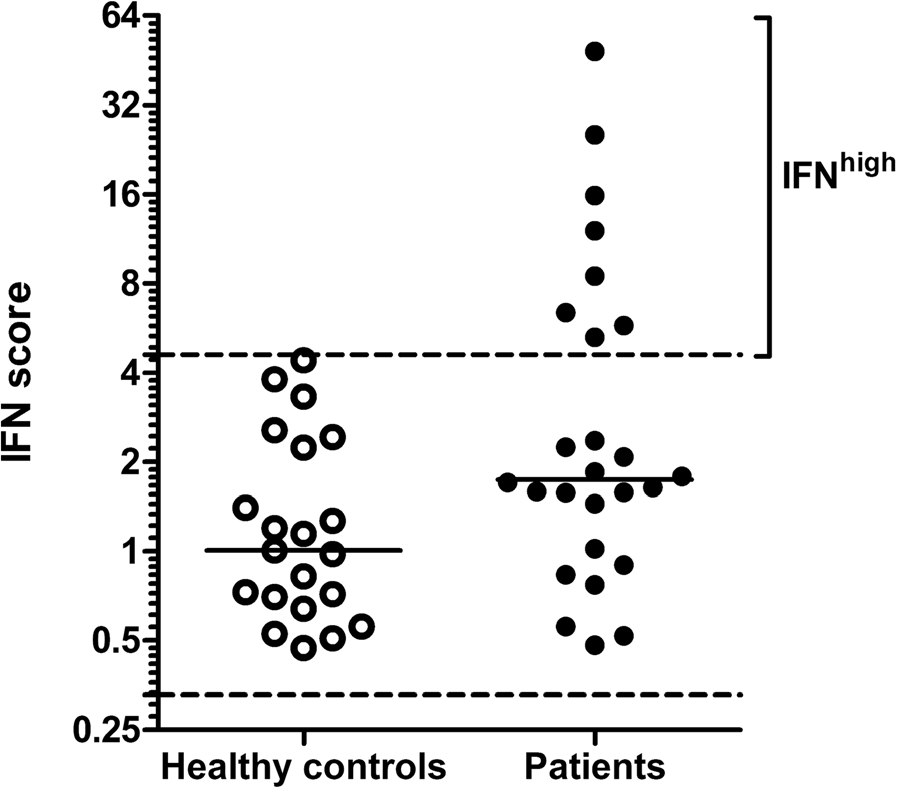
Whole-blood interferon (IFN) scores in all 26 patients with early arthritis. Eight patients were designated patients with an interferon signature (IFN^high^) on the basis of the mean + 2 SD cutoff in healthy control subjects. Patients within the 95 % limits of healthy control subjects (indicated between the *dashed lines*) were considered IFN^low^

First, patients were separated into IFN^high^ and IFN^low^ groups on the basis of their IFN scores in whole blood. As displayed in Fig. 1, the IFN signature was present in 8 of 26 patients, who are referred to as “IFN^high^”; the remaining 18 patients were designated “IFN^low^.” The patients’ characteristics are shown in Table 1. The IFN^high^ group displayed a slightly shorter duration of symptoms and a higher percentage who were ACPA-positive, but this did not reach statistical significance (symptom duration *p* = 0.137, ACPA positivity *p* = 0.084; Fisher’s exact test).

**Table 1:** Patient characteristics

	Healthy control subjects	All patients	IFN^low^	IFN^high^
Number of patients	25	26	18	8
Female, *n* (%)	16 (64)	20 (77)	13 (72)	7 (88)
Age, years, mean (SD)	35 (10)	47 (14)	48 (16)	44 (9)
DAS28, mean (SD)	n/a	4.6 (1.2)	4.7 (1.3)	4.4 (1.0)
Duration of symptoms in weeks,^a^ mean (SD)	n/a	16 (25)	20 (29)	8 (8)
IgM-RF positivity, *n* (%)	n/a	19 (73)	13 (72)	6 (75)
ACPA positivity, *n* (%)	n/a	15 (58)	8 (44)	7 (88)

*Abbreviations: IgM-RF* immunoglobulin M rheumatoid factor, *ACPA* anticitrullinated protein antibodies, *DAS28* Disease Activity Score in 28 joints, *n/a* not applicable, *IFN* ^*low*^ patients within the 95 % limits of healthy control subjects, *IFN* ^*high*^ patients with an interferon signature

^a^Data missing for one patient

### General abundance of cell subsets in relation to whole-blood type I IFN profile

In order to gain insight into the cell subset composition of the peripheral blood in relation to the presence of the IFN signature, we compared the number of total CD3^+^ T cells, CD4^+^ T helper cells, CD8^+^ cytotoxic T cells, CD19^+^ B cells, CD14^+^ monocytes, and granulocytes (PMNs) between IFN^high^ and IFN^low^ patients. As shown in Table 2, we observed a tendency toward lower numbers of all lymphocyte subsets in IFN^high^ patients than in IFN^low^ patients, but this did not reach statistical significance (*p* ≥ 0.07). Remarkably, only the number of PMNs was significantly higher in IFN^high^ patients than in IFN^low^ patients, (1.6-fold, *p* = 0.031). The cell percentages also displayed a slightly higher PMN percentage and lower lymphocyte percentage in IFN^high^ patients than in IFN^low^ patients, although this was not significant (Table 2). The fold difference we observed in whole-blood IFN scores between IFN^low^ patients and IFN^high^ patients (12-fold) greatly exceeded the fold difference observed in PMN abundance (1.6-fold), indicating that the presence of the IFN signature in these patients was not caused primarily by predominance of a particular cell subset.

**Table 2:** Abundance of leukocytes and subsets in patient whole blood

	Based on	All patients	IFN^low^	IFN^high^	Fold difference between means	Comparison of means, *p* value
Total leukocytes	CD45^+^	7191 ± 2626	7271 ± 2981	7020 ± 1814	1.04 (low > high)	0.798
Lymphocytes	CD45^+^, FSC/SSC	1856 ± 481 (27.8 % ± 9.7)	1955 ± 478 (29.7 % ± 10.6)	1646 ± 442 (24.0 % ± 6.1)	1.19 (low > high)	0.194
T cells	CD3^+^	1349 ± 365 (20.2 % ± 7.2)	1441 ± 363 (21.8 % ± 7.8)	1153 ± 304 (16.9 % ± 4.6)	1.25 (low > high)	0.066
Helper T cells	CD3^+^, CD4^+^	850 ± 228 (13.1 % ± 5.0)	899 ± 214 (13.8 % ± 5.3)	729 ± 229 (11.6 % ± 4.1)	1.23 (low > high)	0.130
Cytotoxic T cells	CD3^+^, CD8^+^	465 ± 232 (6.9 % ± 3.4)	514 ± 245 (7.6 % ± 3.7)	345 ± 148 (5.2 % ± 1.9)	1.49 (low > high)	0.187
B cells	CD19^+^	268 ± 125 (4.0 % ± 1.9)	284 ± 140 (4.2 % ± 2.1)	234 ± 81 (3.5 % ± 1.3)	1.21 (low > high)	0.549
Monocytes	CD14^+^	336 ± 127 (4.8 % ± 1.5)	353 ± 140 (5.0 % ± 1.4)	299 ± 92 (4.4 % ± 1.5)	1.18 (low > high)	0.406
PMNs	FSC/SSC	3757 ± 2715 (67.4 % ± 10.1)	3137 ± 2945 (65.3 % ± 11.0)	5075 ± 1598 (71.6 % ± 6.7)	1.61 (low < high)	0.031

*Abbreviations: FSC* forward scatter SSC, side scatter, *IFN* ^*low*^ patients within the 95 % limits of healthy control subjects, *IFN* ^*high*^ patients with an interferon signature, *PMN* polymorphonuclear granulocyte

Cell amounts are indicated in numbers per microliter, mean ± SD. Percentages of total leukocytes are indicated between brackets, mean ± SD

### Contribution of sorted cell subsets to IFN score

**Fig. 2:**
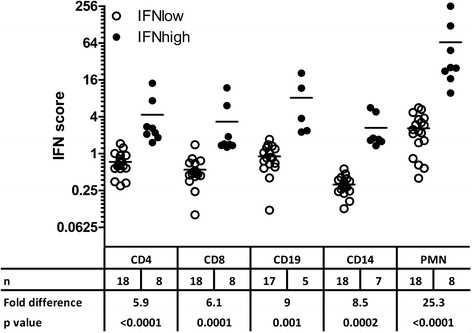
Interferon (IFN) scores per leukocyte subset of patients within the 95 % limits of healthy control subjects (IFN^low^) and patients with an interferon signature (IFN^high^). Fold differences between the two groups, as well as *p* values of the statistical comparisons, are indicated below the graph. *PMN* polymorphonuclear granulocyte

Next, we compared the contribution of individual leukocyte subsets to the IFN signature. As shown in Fig. 2, IFN scores were significantly different between IFN^high^ and IFN^low^ patients for all cell subsets, which is to be expected because all cell types presumably possess type I IFN signaling ability. The difference between IFN^high^ and IFN^low^ patients was most prominent for the PMN fraction, which displayed a 25-fold higher mean IFN score in IFN^high^ patients than in IFN^low^ patients (*p* < 0.0001) (Fig. 2). These measurements are normalized to RNA input, and the expression levels are relative to the housekeeping gene *GAPDH*; hence, these data are without regard to cell abundance.

In order to investigate the relative contribution of the leukocyte subsets in relation to their distribution in peripheral blood, we used the expression data in whole blood from IFN^high^ patients and the relative abundance of each subset to estimate an “expected” cell subset contribution, assuming that each subset would contribute equally to the IFN score. Subsequently, we compared the estimated cell subset contributions to the actual contributions as measured in the sorted cell subsets, corrected for the cell subset's abundance (“observed” contribution). Details about the calculation of the expected and observed contributions are described above in the Methods section.

As shown in Table 3, all cell types showed a difference between the observed contributions and the expected contributions to the IFN score. The observed contributions of CD4^+^ helper T cells, CD8^+^ cytotoxic T cells, CD19^+^ B cells, and CD14^+^ monocytes were 2.8- to 6.3-fold less than the expected contributions, which was significant for most subsets (*p* ≤ 0.0625). Remarkably, the observed contribution of the PMNs was 3.4-fold greater than its expected contribution (*p* = 0.0078). This tendency remained present after correction for any differences in RNA yield between subsets (data not shown). The sum of the RNA-corrected observed contributions of all cell subsets per patient was somewhat higher than the total IFN score as measured in whole blood (mean difference 1.2-fold, not significantly different). This could be explained by slight impurities in each isolated subset, and it implies that there is no other cell population substantially contributing to the whole-blood IFN score, as this would have resulted in a lower sum than the whole-blood IFN score. Altogether, these data indicate that PMNs are the main contributors to the whole-blood IFN score, not only due to its high abundance in whole blood but also because of an increased potency to induce IRGs.

**Table 3:** Expected and observed contributions of leukocyte subsets to the interferon score in whole blood of patients with an interferon signature

	Expected	Observed	Direction	Mean fold difference	*p* Value
CD4	1.843 ± 1.715	0.521 ± 0.507	Exp > Obs	3.94 ± 1.71	0.0156
CD8	0.741 ± 0.504	0.156 ± 0.127	Exp > Obs	5.36 ± 2.90	0.0223
CD19	0.530 ± 0.499	0.298 ± 0.263	Exp > Obs	2.78 ± 0.68	0.0625
CD14	0.679 ± 0.594	0.126 ± 0.110	Exp > Obs	6.25 ± 2.53	0.0156
PMNs	11.71 ± 11.53	48.33 ± 65.89	Exp < Obs	3.35 ± 1.29	0.0078

*Abbreviations: Exp* expected, *Obs* observed, *PMN* polymorphonuclear granulocyte

### Relation between IFN score and type I IFN receptor expression in subsets and whole blood

The data described above suggests an increased sensitivity of IFN^high^ PMNs to type I IFNs. To gain more insight into the mechanism behind this increased sensitivity, we measured the mRNA expression of the upstream receptors of type I IFN signaling (i.e., *IFNAR1* and *IFNAR2*). Although we observed a correlation between the subset’s IFN score and *IFNAR1* expression for all subsets, the correlation between the subset’s IFN score and *IFNAR2* expression was significant only for the PMN fraction (Spearman’s *r* = 0.461, *p* = 0.020) (Table 4). Furthermore, both *IFNAR1* and *IFNAR2* expression were highest in the PMN fraction compared with the other fractions, indicating that PMNs could be more sensitive to type I IFN binding.

**Table 4:** Type I interferon receptors *IFNAR1* and *IFNAR2* mRNA expression and their relationship to subset interferon scores

	Average expression		Correlation with subset’s IFN score	
	All patients	IFN^high^ patients	Spearman’s *r*	*p* Value
*IFNAR1*				
CD4	2.473 ± 1.157	3.440 ± 1.309	0.679	0.0001
CD8	1.723 ± 0.701	1.976 ± 0.730	0.363	0.069
CD19	3.687 ± 1.756	3.844 ± 1.487	0.525	0.012
CD14	0.687 ± 0.294	0.908 ± 0.354	0.371	0.068
PMN	5.089 ± 2.243	6.529 ± 1.649	0.461	0.020
*IFNAR2*				
CD4	4.544 ± 3.247	3.531 ± 1.234	0.179	0.382
CD8	3.952 ± 2.736	2.969 ± 0.994	−0.037	0.857
CD19	4.213 ± 2.389	2.978 ± 0.908	−0.03	0.895
CD14	0.946 ± 0.562	0.748 ± 0.304	0.042	0.842
PMN	5.749 ± 5.039	6.231 ± 3.340	0.507	0.010

*Abbreviations: IFN* interferon, *IFNAR* interferon α/β receptor, *IFN* ^*high*^ patients with an interferon signature, *PMN* polymorphonuclear granulocyte

### Specific upregulation of type I IFN receptors in early arthritis PMNs

**Fig. 3:**
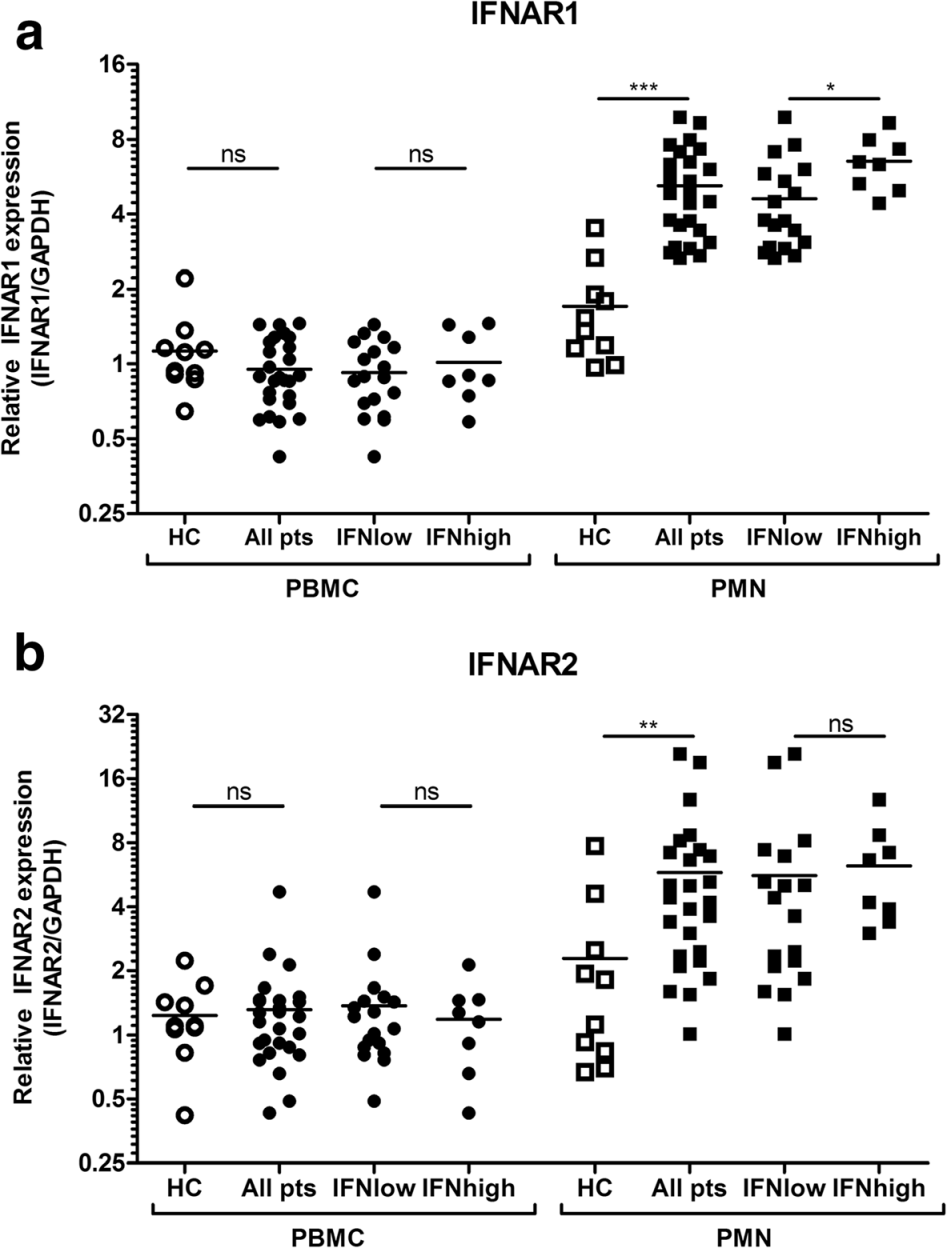
Selective upregulation of interferon α/β receptor (*IFNAR*) mRNA expression in polymorphonuclear granulocytes (PMNs) from patients with early arthritis. *IFNAR1* (**a**) and *IFNAR2* (**b**) expression in peripheral blood mononuclear cells (PBMCs) and PMNs from healthy control subjects (HC) and patients with early arthritis. *p = 0.05–0.01 **p = 0.01–0.001; ***p ≤ 0.001. *IFN* ^*high*^ patients with an interferon signature, *IFN* ^*low*^ patients within the 95 % limits of healthy control subjects, *ns* not significant

Since the PMN fraction showed a high activation of the IFN response that appeared to be related to expression of the type I IFN receptors, we compared *IFNAR1* and *IFNAR2* mRNA expression in isolated PBMCs and PMNs of patients with those of healthy control subjects. As shown in Fig. 3, we observed no differences in expression of *IFNAR1* or *IFNAR2* in the PBMC fractions of patients compared with healthy control PBMCs (*p* = 0.387 and *p* = 0.902, respectively). However, both *IFNAR1* and *IFNAR2* expression were considerably increased in the PMN fraction of patients compared with healthy control PMNs (IFNAR1 3.0-fold, *p* < 0.001; IFNAR2 2.5-fold, *p* = 0.008). Only *IFNAR1* expression was significantly different between IFN^low^ and IFN^high^ patients (*p* = 0.021) (see Fig. 3a), implying that the extent of the IFN signature might not depend solely on *IFNAR* expression.

## Discussion

The type I IFN signature in peripheral blood from patients with RA was first described in 2007 [1]; since then, it has been studied extensively in relation to disease onset and therapeutic response. Occasionally, IRG expression in RA was assessed in isolated cell subsets instead of in whole blood, such as PBMCs [6, 19], monocytes [20], or neutrophils [21]. To our knowledge, the present study is the first to demonstrate that there is diversity in the contribution of whole-blood cell subsets to the extent of the type I IFN response, with a major contribution by PMNs.

Patients with an IFN signature (IFN^high^) did not appear clinically different from patients without this signature (IFN^low^). Although our cohort is rather small, the results corroborate those of previous studies [1, 3]. We observed slightly lower lymphocyte counts and slightly higher neutrophil counts in IFN^high^ patients than in IFN^low^ patients, but these differences were too small to fully explain the difference in whole-blood IFN scores between the two groups. Concordantly, our data suggest that the whole-blood IFN signature is facilitated by a selective change in PMN sensitivity to type I IFN signaling rather than by a great difference in cell abundance.

The PMN fraction consists primarily of neutrophils, which have been shown to play a role in RA. They are the first cells to enter the joint when the disease starts and are the most abundant cell type present in the joint [22, 23]. Neutrophils in the RA joint display a “primed” phenotype compared with control neutrophils, resulting in increased cytokine and chemokine production, decreased apoptosis rates [24], a gained ability to present antigens [25], and upregulation of chemokine receptors to induce migration of other immune cells [26].

We observed that patient PMNs, but not PBMCs, displayed type I IFN receptor (*IFNAR1* and *IFNAR2*) upregulation compared with healthy control subjects, which was not completely dependent on the presence of the IFN signature. It has been suggested that RA neutrophils would mainly become primed and activated within the inflamed joint due to the large amount of cytokines present. However, the *IFNAR1* and *IFNAR2* upregulation in the circulating PMNs suggests that these cells could also have gained a primed phenotype. Wright and colleagues described the gene profiles that are induced upon neutrophil priming with TNF-α or granulocyte-macrophage colony-stimulating factor, which did not involve upregulation of *IFNAR1* and even seemed to cause downregulation of *IFNAR2* [27]. Broader gene expression and protein expression studies on RA PMNs, possibly paired with synovial PMNs, are required to gain more insight into the exact gene profile and source of the priming.

It was demonstrated that healthy mature neutrophils already display increased expression of *IFNAR1* and *IFNAR2* as well as type I IFN response genes compared with immature neutrophils [28]. Of interest, these mature neutrophils were more prone to IFN-α-mediated induction of neutrophil extracellular trap (NET) formation than immature neutrophils. NETs are extracellular structures that consist of chromatin and neutrophil-related proteins and are released by neutrophils under (auto-)inflammatory conditions. Neutrophils from RA blood and synovial fluid have been shown to exhibit increased spontaneous NET formation compared with neutrophils from healthy control subjects [29, 30] or patients with osteoarthritis [31]. A study of patients with systemic lupus erythematosus demonstrated that NETs contain a considerable source of type I IFN-inducing agents [32]. Altogether, the upregulation of *IFNAR1* and *IFNAR2* we observed in RA PMNs, together with the increased spontaneous NET formation, could contribute to a positive feedback loop of subsequent NET-mediated type I IFN production, type I IFN binding, and simultaneous IRG induction and more NET formation.

It was recently demonstrated that the baseline IFN signature in RA PMNs is associated with a good response to anti-TNF therapy [21]. Notably, in earlier studies using gene expression profiling in whole blood, researchers described a relationship only between IFN response regulation and therapeutic response during anti-TNF treatment and not between the extent of the IFN response and anti-TNF response prior to the start of therapy [11, 12]. Although the researchers in these previous studies described different types of anti-TNF treatment and the PMN findings need validation in independent studies, one could speculate that the PMN fraction is a more homogeneous source than whole blood to study the IFN signature in relation to anti-TNF response. Moreover, neutrophils are known to both bind and secrete TNF-α, and multiple studies have demonstrated that TNF-α and type I IFNs might influence each other’s signaling activities [33, 34]. Consequently, the IFN signature in PMNs might be a direct reflection of high TNF-α activity and therefore indicate increased sensitivity to TNF-α inhibition, ultimately resulting in a good response to therapy.

The presence of a baseline IFN signature was also shown to be associated with a poor response to rituximab treatment [6, 7]. The exact mechanism behind this association remains to be elucidated, but it could indicate that patients with an IFN signature have a neutrophil-dominated pathology and that hence B-cell depletion would have less effect on disease activity than in IFN^low^ patients. Recently, it was shown that rituximab treatment could lead to late-onset neutropenia in a small proportion of patients [35]. It would be interesting to study this in relation to the previously reported association of rituximab-related pharmacodynamics of type I IFN response gene expression and clinical response to rituximab [10].

Considering these previously described findings regarding the IFN signature in relation to therapy response, we hypothesize that patients with an IFN signature in the neutrophils might benefit from therapies that target the activity of neutrophil-derived cytokines, such as anti-TNF therapy [21] or tocilizumab therapy [9], whereas patients without an IFN signature might benefit from rituximab therapy instead [6, 7]. However, more studies on the exact role of the IFN signature in neutrophil-related RA pathology are required to support this hypothesis.

PMNs are considered one of the first cell types to enter the joint [22], and the presence of an IFN signature has been associated with an increased risk to develop arthritis [4, 5], which could indicate that the neutrophils have been primed and activated to migrate toward the joint in order to inflict the first damage. Moreover, it could suggest that patients without an IFN signature who develop arthritis might have another mechanism behind the disease’s onset, such as mediation by B-cell migration [4, 36–38]. Extending the present study to the preclinical phase of arthritis could develop more insight into the role of the IFN signature and neutrophils in disease onset.

## Conclusions

We have conclusively demonstrated that PMNs are the main contributors to the whole-blood IFN signature in patients with early arthritis. Considering the well-established role of neutrophils in the pathology of arthritis, this suggests a role of type I IFN activity in the disease as well.

## Abbreviations

ACPA, anticitrullinated protein antibodies; APC, allophycocyanin; cDNA, complementary DNA; DAS28, Disease Activity Score in 28 joints; FITC, fluorescein isothiocyanate; FSC, forward scatter; HC, healthy controls; IFN, interferon; IFNAR, interferon α/β receptor; IFN^high^, patients with an interferon signature; IFN^low^, patients within the 95 % limits of healthy control subjects; IgM-RF, immunoglobulin M rheumatoid factor; IRG, interferon response gene; JAK, Janus kinase; mRNA, messenger RNA; NET, neutrophil extracellular trap; PBMC, peripheral blood mononuclear cell; PE, phycoerythrin; PerCP, peridinin chlorophyll; PMN, polymorphonuclear granulocyte; qPCR, quantitative polymerase chain reaction; RA, rheumatoid arthritis; SSC, side scatter; STAT, signal transducer and activator of transcription; TNF, tumor necrosis factor

## Additional file

Additional file 1: Table S1. List of primers used for quantitative PCR. (PDF 94 kb)
