# Supplementary material for: The type I interferon signature in leukocyte subsets from peripheral blood of patients with early arthritis: a major contribution by granulocytes
Source: Arthritis Res Ther. 2016 Jul 13;18:165. doi: 10.1186/s13075-016-1065-3 (PMC4944477; doi:10.1186/s13075-016-1065-3)
Supplement: Additional file 1: Table S1. — List of primers used for quantitative PCR. (PDF 94 kb) [file 13075_2016_1065_MOESM1_ESM.pdf]

Table S1: Primers used for quantitative PCR		
**Gene of interest**	**Forward primer**	**Reverse primer**
GAPDH	GCCAGCCGAGCCACATC	TGACCAGGCGCCCAATAC
RSAD2	GTGGTTCCAGAATTATGGTGAGTATTT	CCACGGCCAATAAGGACATT
IFI44L	CCGAGCGGTATAGGATATATTCTGTT	TGCTCCTTCTGCCCCATCTA
MX1	TTCAGCACCTGATGGCCTATC	GTACGTCTGGAGCATGAAGAACTG
IFNAR1	AAAATTGTCTGGGTGTCAGAATATTACTAG	ACCAATCTGAGCTTTGCGAAA
IFNAR2	TGTATACAATCATGAGTAAACCAGAAGATTT	TTGTGTTCCCGCTGAATCCT
